# Low Resistance Asymmetric III-Nitride Tunnel Junctions Designed by Machine Learning

**DOI:** 10.3390/nano11102466

**Published:** 2021-09-22

**Authors:** Rongyu Lin, Peng Han, Yue Wang, Ronghui Lin, Yi Lu, Zhiyuan Liu, Xiangliang Zhang, Xiaohang Li

**Affiliations:** 1 Advanced Semiconductor Laboratory, King Abdullah University of Science and Technology, Thuwal 23955, Saudi Arabia; rongyu.lin@kaust.edu.sa (R.L.); yue.wang@kaust.edu.sa (Y.W.); ronghui.lin@kaust.edu.sa (R.L.); yi.lu@kaust.edu.sa (Y.L.); zhiyuan.liu@kaust.edu.sa (Z.L.); 2 Laboratory Machine, Intelligence and kNowledge Engineering (MINE), King Abdullah University of Science and Technology, Thuwal 23955, Saudi Arabia; peng.han@kaust.edu.sa; 3 Department of Computer Science and Engineering, University of Notre Dame, Notre Dame, IN 46556, USA

**Keywords:** tunnel junction, machine learning, III-nitride

## Abstract

The tunnel junction (TJ) is a crucial structure for numerous III-nitride devices. A fundamental challenge for TJ design is to minimize the TJ resistance at high current densities. In this work, we propose the asymmetric p-AlGaN/i-InGaN/n-AlGaN TJ structure for the first time. P-AlGaN/i-InGaN/n-AlGaN TJs were simulated with different Al or In compositions and different InGaN layer thicknesses using TCAD (Technology Computer-Aided Design) software. Trained by these data, we constructed a highly efficient model for TJ resistance prediction using machine learning. The model constructs a tool for real-time prediction of the TJ resistance, and the resistances for 22,254 different TJ structures were predicted. Based on our TJ predictions, the asymmetric TJ structure (p-Al_0.7_Ga_0.3_N/i-In_0.2_Ga_0.8_N/n-Al_0.3_Ga_0.7_N) with higher Al composition in p-layer has seven times lower TJ resistance compared to the prevailing symmetric p-Al_0.3_Ga_0.7_N/i-In_0.2_Ga_0.8_N/n-Al_0.3_Ga_0.7_N TJ. This study paves a new way in III-nitride TJ design for optical and electronic devices.

## 1. Introduction

The use of tunnel junction (TJ) is crucial for many advanced III-nitride electronic and optical devices, including tunnel field-effect transistors (TFETs), light-emitting diodes (LEDs), and solar cells [1,2,3]. For instance, TJs could replace the use of resistant and absorptive p-type layers and contacts in UV-LEDs. In addition, TJs enable cascading optoelectronic devices, which provide greater flexibility for functional designs [4]. A significant polarization effect provides more space to manipulate the performance of TJs [5]. Recently, GaN/AlGaN/GaN [6,7], metal/InGaN/GaN [8], and GaN/InGaN/GaN [9,10,11] TJs were investigated and proven to lead to significant improvements in devices.

The interplay of material compositions, polarization effects, and the thicknesses of each layer in the TJ structures provide enormous design parameter space for TJ designs, which increases the difficulties associated with TJ optimization. To this end, the Technology Computer-Aided Design (TCAD) software has been widely employed to validate TJ effectiveness [11,12,13,14]. However, massive calculations are required to investigate the enormous possibilities of TJ designs. In addition, the convergence issue caused by the self-consistent solution of classical and quantum equations has made a systematic investigation of TJ design even more difficult. Currently, there are no effective methods to circumvent these issues except the cumbersome trial-and-error method. Recently, the machine-learning (ML) technique has demonstrated its significant effectiveness to these TCAD convergence issues including for III-nitride LED and nanophotonics designs [15,16,17,18]. It is a global optimization tool that can search the whole parameter space and yield a viable design efficiently. We demonstrate, for the first time, machine learning can be applied to alleviate the above-mentioned issues in the designing of III-nitride TJs significantly. Although the computational resources are required at the development stage of the algorithm, it is a one-time cost and once the algorithm is developed, the algorithm runs on very few resources in real time without convergence issues.

The symmetric p-AlGaN/i-InGaN/n-AlGaN tunnel junctions have been widely applied in LEDs and HEMTs (high-electron-mobility transistors) for enhancing device performances [19,20,21,22]. The TJs are usually assumed with the same Al composition in both p-AlGaN and n-AlGaN layers without a detailed discussion of this symmetryA. Although the AlGaN grading designs with the change of the Al composition in p-layer or n-layer were introduced to enhance the device performance [2], it did not explain how the asymmetric Al composition in the p-AlGaN and n-AlGaN influence the p-AlGaN/i-InGaN/n-AlGaN TJ resistance.

In this work, we employed ML to explore the asymmetric p-AlGaN/i-InGaN/n-AlGaN TJ design by predicting TJ resistance. The XG-Boost strategy [23] was leveraged to directly predict the TJ resistance. We trained the ML model using cross-validated results by TCAD software with different TJ configurations. To exclude the outliers caused by the convergence issues, all the configurations were calculated twice with two different iterations and cross-validated by the results. The accuracy of the model is 90.5% in the test datasets, which suggested its great capacity to predict TJ resistance. We also used this model to generate 22,254 TJ configurations. Afterward, we investigated the configurations with relatively low resistance; and we discovered that the asymmetric TJ design such as p-Al_0.7_Ga_0.3_N/i-In_0.2_Ga_0.8_N/n-Al_0.3_Ga_0.7_N with different Al compositions in the p-type and n-type layers, could lead to considerably lower (about seven times) TJ resistance compared with conventional symmetric p-Al_0.3_Ga_0.7_N/i-In_0.2_Ga_0.8_N/n-Al_0.3_Ga_0.7_N design.

## 2. TCAD Calculations of p-AlGaN/i-InGaN/n-AlGaN TJ

**Figure 1:**
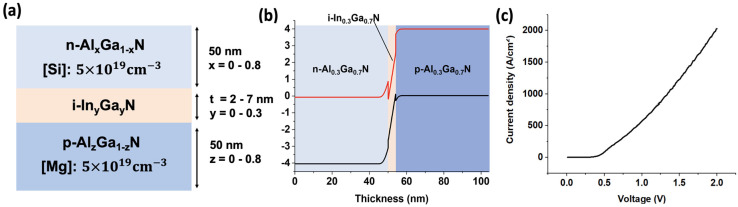
(**a**) Cross-sectional schematics of the proposed p-Al_x_Ga_1-x_N/i-In_y_Ga_1-y_N/n-Al_z_Ga_1-z_N TJ structures. (**b**) An example of the p-Al_0.3_Ga_0.7_N/i-In_0.3_Ga_0.7_N/n-Al_0.3_Ga_0.7_N TJ band structure. (**c**) Calculated current–voltage curve for the p-Al_0.3_Ga_0.7_N/i-In_0.3_Ga_0.7_N/n-Al_0.3_Ga_0.7_N tunnel junction.

We investigated the p-Al_x_Ga_1-x_N/i-In_y_Ga_1-y_N/n-Al_z_Ga_1-z_N TJs as shown in Figure 1a. Four features, including the Al compositions x and z of the p-Al_x_Ga_1-x_N and n-Al_z_Ga_1-z_N layers, the In composition y of the i-In_y_Ga_1-y_N layer, and the thickness t of the i-In_y_Ga_1-y_N layer, are selected to describe the TJ configurations. The doping concentrations of [Mg] and [Si] were set to be $5\times 10^{19}$ cm^−3^ and the In composition of the thin i-InGaN layer was in the range of 0–0.3, which was achievable in most growth systems [5,24]. The Al compositions were in the range of 0–0.8. We assumed a linear change in the acceptor activation energy from 140 meV in GaN to 630 meV in AlN, and a constant donor activation energy of 15 meV for AlGaN with an Al composition lower than 80% [25,26]. As shown in Figure 1b, because the band structures of p-AlGaN and n-AlGaN move into the flat band zone from the interface within 20 nm (usually less than 10 nm), we set the p-AlGaN and n-AlGaN layer to be 50 nm, a typical value which is thick enough for the band structure to form a flat band zone [11]. The thickness of the inserted InGaN layer is set within 2–7 nm [10,27]. This thin InGaN layer could induce a larger polarization charge at the interface, where the smaller bandgap of the InGaN increased the tunneling probability [9,10,11]. We carried out the TCAD calculations using Silvaco Atlas, a two-dimensional (2D) device simulator by consistently solving Schrodinger–Poisson equations, and used a non-local band-to-band tunneling model to calculate the TJ resistance [28]. The calculations were all conducted at a current density of 10 A/cm^2^ for a fair comparison, which is a typical order in III-nitride tunnel junction research [2]. An example of a calculated current–voltage curve for the p-Al_0.3_Ga_0.7_N/i-In_0.3_Ga_0.7_N/n-Al_0.3_Ga_0.7_N tunnel junction is shown in Figure 1c. The strain is considered in the polarization calculation, and the polarization parameters, electronic band structures, mobility parameters and other materials properties not mentioned above were using the default values recorded in ref. [28].

We collected approximately 3500 results from the TCAD calculations. Because the convergence issue that occurs in the calculation may cause some data error, we calculated all the configurations twice with different iteration steps. These convergence issues usually lead to abrupt changes in the I–V curve [28]. This means that the corresponding resistances would suffer from convergence issues that usually exhibited a significant deviation from the other calculations according to the different iteration steps. Using this cross-validation method, we effectively ruled out the unreliable data that may have had a negative impact on the ML model and reduced the accuracy of the model prediction.

Specifically, we cross-validated the calculated data as credible when two calculation results had a data verification difference that was less than 5%, according to the following formula:

(1) $$\frac{\left|\mathrm{TJ}\;\mathrm{resistance}\;\mathrm{data}\;1-\mathrm{TJ}\;\mathrm{resistance}\;\mathrm{data}\;2\right|}{\mathrm{TJ}\;\mathrm{resistance}\;\mathrm{data}\;1}=\{\begin{array}{c} \left(<5\%\;(\mathrm{credible},\;\mathrm{no}\;\mathrm{convergence}\;\mathrm{issue})\right)\\ \left(>5\%\;(\mathrm{false},\;\mathrm{suffered}\;\mathrm{from}\;\mathrm{convergence}\;\mathrm{issue})\right) \end{array}$$

After cross-validation, we had 2260 remaining data points, which were used to construct the TJ resistance database for the ML model. Considering the calculation resources, we set the iteration step as 0.01 Ω/cm^2^ and set the terminated value of the maximum TJ resistance as 100 Ω/cm^2^.

## 3. Machine Learning Model for TJ Resistance Prediction

**Figure 2:**
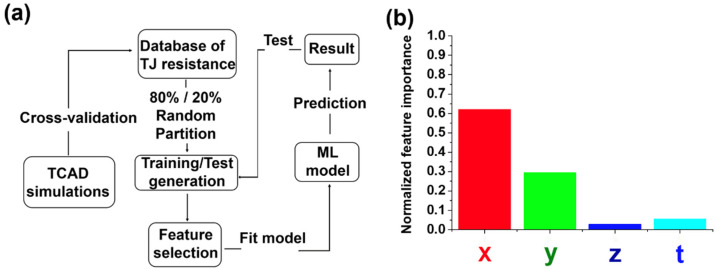
(**a**) Process flow of the ML model for TJ resistance prediction. (**b**) Feature importance retrieved from the XG-Boost model that was learned from the 2260 data samples.

Subsequently, the ML model was applied to the TJ resistance dataset. The schematic representation of the proposed ML model is shown in Figure 2a. By random partition, we used 80% of the dataset as the training data and considered the remaining to be the test data. We selected the XG-Boost classifier as the model [23], which is a boosting method that is widely used in many applications. Unlike many learning methods which have a fixed objective function and parameter format, the objective function and the format of the parameter in XG-Boost change iteratively in the process of optimization. For every iteration, the objective function of XG-Boost is to minimize the residuals of the last iteration. In this study, given the corresponding features $\left\{x_{1},x_{2},\ldots ,x_{n}\right\}$ and the results $\left\{y_{1},y_{2},\ldots ,y_{n}\right\}$, the objective function of the first iteration in XG-Boost is as follows:

(2) $$Obj^{\left(1\right)}=\sum_{i=1}^{n}l\left(y_{i},f_{1}\left(x_{i}\right)\right)+\Omega \left(f_{1}\right),$$

$l\left(y_{i},y_{k}\right)$ is the difference function between $y_{i}$ and $y_{k}$; we set $l\left(y_{i},y_{k}\right)={\left(y_{i}-y_{k}\right)}^{2}$; and $\Omega \left(f_{1}\right)$ is the regularization term of function $f_{1}$, which is used to overcome the overfitting problem. Once we minimized $Obj^{\left(1\right)}$ to get the function $f_{1}$, we used ${\hat{y}}_{i}^{\left(1\right)}=f_{1}\left(x_{i}\right)$ to denote the predicted result of the i-th feature. The second iteration of XG-Boost is to minimize the residuals of the first iteration, as follows:

(3) $$Obj^{\left(2\right)}=\sum_{i=1}^{n}l\left(y_{i},{\hat{y}}_{i}^{\left(1\right)}+f_{2}\left(x_{i}\right)\right)+\Omega \left(f_{2}\right).$$

Generally, for the *t* + 1-th iteration, we used

(4) $$Obj^{\left(n+1\right)}=\sum_{i=1}^{n}l\left(y_{i},{\hat{y}}_{i}^{\left(t\right)}+f_{t+1}\left(x_{i}\right)\right)+\Omega \left(f_{t+1}\right),$$

where ${\hat{y}}_{i}^{\left(t\right)}=\overset{t}{\underset{j=1}{\sum }}f_{j}\left(x_{i}\right)$ and ${\hat{y}}_{i}^{\left(0\right)}=0$. Once we finished the training process of XG-Boost with *t* iterations, we obtained the final predicted $y_{i}^{p}$ of the *i*-th feature as the sum of all prediction functions as $y_{i}^{p}={\hat{y}}_{i}^{\left(T\right)}=\overset{T}{\underset{j=1}{\sum }}f_{j}\left(x_{i}\right)$.

Under this framework, XG-Boost has better generalization and representation ability with a dense dataset, in which the number of samples is much larger than the dimension of features. By contrast, a sparse dataset easily leads to the overfitting problem [29]. To generalize our model extensible to the sparse dataset, a regularization term is added in the objective function to overcome the overfitting problem. Moreover, the computation of different functions in the inference process could be applied in parallel, which reduces the time complexity. The high average testing performance proves the feasibility of applying ML to TJ resistance prediction. Our predictions demonstrate that the XG-Boost model gives an average accuracy of 88.7%, with 90.5% accuracy being the best. We considered the prediction result to be true when it had an error of less than 10%.

**Figure 3:**
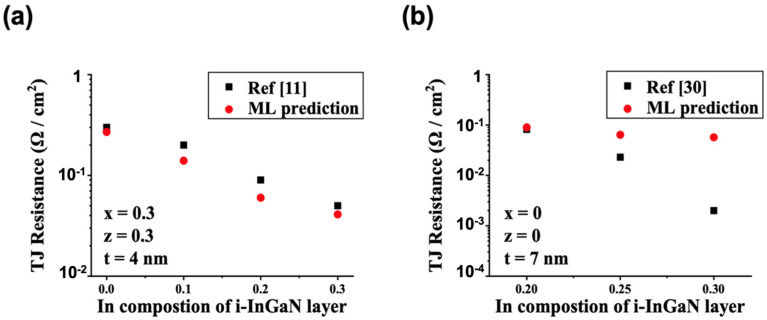
(**a**) p-Al_0.3_Ga_0.7_N/i-In_y_Ga_1-y_N/n-Al_0.3_Ga_0.7_N TJ structures with the 4 nm interlayer [11], and (**b**) p-GaN/i-In_y_Ga_1-y_N/n-GaN TJ structures with the 7 nm interlayer [30].

The importance of the four features x, y, z, t could be extracted from the XG-Boost model. In Figure 2b, the Al composition of n-AlGaN and the In composition of the InGaN layer play the most important roles for TJ resistance optimization. To further verify our data, we compared our TJ resistance prediction results according to the proposed model. Results are shown in Figure 3.

As shown in Figure 3a, our ML prediction for p-Al_0.3_Ga_0.7_N/i-In_y_Ga_1-y_N/n-Al_0.3_Ga_0.7_N TJ structures with the 4 nm interlayer agreed well with the previous report [11]. Figure 3b shows the p-GaN/i-In_y_Ga_1-y_N/n-GaN TJ structures with the 7 nm interlayer. Although the TJ resistances when In equals 0.2 and 0.25 predicted by ML agree with previous reports [30], it shows a relatively large deviation from earlier reference when In equals 0.3. The iteration step in our TCAD simulation was 0.01 Ω/cm^2^, which meant that a TJ resistance less than 0.01 Ω/cm^2^ could not be accurately recorded. This may have caused the inaccuracy in the exact TJ resistance value for the ultra-low TJ resistance configuration. The tendency of the TJ resistance variation, however, was still meaningful for TJ design instruction. In addition, we believed that the prediction could be improved significantly by reducing the iteration step and by using more powerful calculation resources.

## 4. TJ Resistance Prediction Results

**Figure 4:**
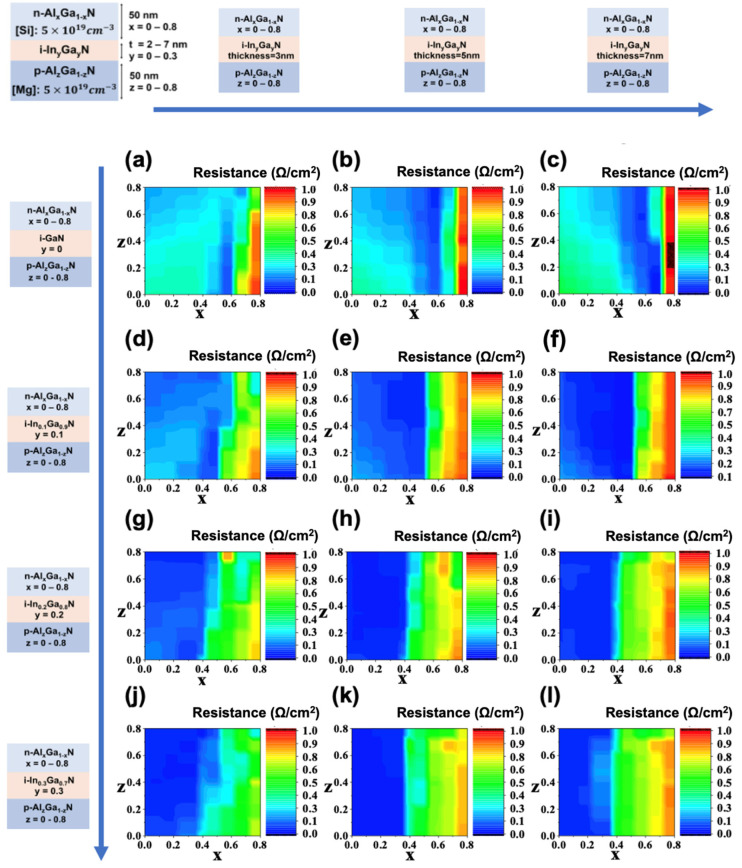
p-Al_x_Ga_1-x_N/i-In_y_Ga_1-y_N/n-Al_z_Ga_1-z_N TJ resistance prediction results with the values of the thickness of i-InGaN layer t and In composition of the i-InGaN layer y: (**a**) t = 3 nm, y = 0; (**b**) t = 5 nm, y = 0; (**c**) t = 7 nm, y = 0; (**d**) t = 3 nm, y = 0.1; (**e**) t = 5 nm, y = 0.1; (**f**) t = 7 nm, y = 0; (**g**) t = 3 nm, y = 0.2; (**h**) t = 5 nm, y = 0.2; (**i**) t = 7 nm, y = 0.2; (**j**) t = 3 nm, y = 0.3; (**k**) t = 5 nm, y = 0.3; (**l**) t = 7 nm, y = 0.3.

Using the ML model, we predicted 22,254 combinations of TJ configurations with different x, y, z, t. The results of some of these configurations are shown in Figure 4. Higher Al composition in the n-AlGaN layer often leads to higher TJ resistance. Higher Al composition in the n-AlGaN leads to larger band discontinuity in the AlGaN/InGaN interface, and the tunneling probability decreases when the band discontinuity increases. By comparing these results with the different rows, i.e., different In compositions in Figure 4, we found that the higher In composition in the i-InGaN layer significantly reduced the TJ resistance, in particular for an Al composition in the n-AlGaN layer larger than 0.6. For higher-Al-composition p-AlGaN/i-InGaN/n-AlGaN TJs, the larger induced polarization charges in the AlGaN/InGaN surfaces reduce the depletion region, which assists the carriers in tunneling through the barriers. The thickness of the InGaN layer is critical in the p-AlGaN/i-InGaN/n-AlGaN TJs. If the InGaN layer is too thin, conduction and valence band extrema on either side do not align with enough region [31]. The extra-thick InGaN is introduced in a resistance series, which may have increased the resistance as well [10].

## 5. Asymmetric TJ with Different Al Compositions in p-AlGaN and n-AlGaN

**Figure 5:**
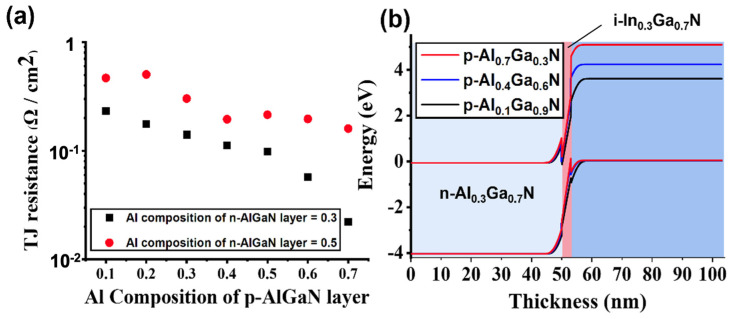
(**a**) TJ resistance prediction of p-AlGaN/i-In_0.2_Ga_0.8_N/n-Al_0.3_Ga_0.7_N and p-AlGaN/i-In_0.2_Ga_0.8_N/n-Al_0.5_Ga_0.5_N with different Al compositions of the p-AlGaN layer. (**b**) Band diagram of p-AlGaN/i-In_0.2_Ga_0.8_N/n-Al_0.3_Ga_0.7_N when the Al composition of the p-AlGaN layer is equal to 0.1, 0.4, and 0.7. The thickness of this i-In_0.2_Ga_0.8_N layer is 3 nm.

Most previous TJ designs used a symmetrical structure, where the Al compositions of the p-AlGaN layer and n-AlGaN layer had the same values [32]. For a large part of the configurations, the effect of adjusting the Al composition of p-AlGaN was not significant. Thus, most studies have ignored the impact of this asymmetry. Our ML model, however, provides a novel asymmetric TJ design. We could identify many examples of the configurations, whose asymmetrical design could significantly reduce the TJ resistance and thus improve device performance. Figure 5a shows TJ resistance predictions for p-AlGaN/i-In_0.2_Ga_0.8_N/n-Al_0.3_Ga_0.7_N and p-AlGaN/i-In_0.2_Ga_0.8_N/n-Al_0.5_Ga_0.5_N with different Al compositions for the p-AlGaN layer. The thickness of the i-In_0.2_Ga_0.8_N layer is 3 nm. We observed that the TJ resistances did not reach their minimum values when the Al composition in p-AlGaN was equal to that in n-AlGaN. For TJs with an n-Al_0.3_Ga_0.7_N layer, in particular, the TJ resistance when the Al composition in p-AlGaN is equal to 0.3 is 0.14 Ω/cm^2^, which is seven times larger than when that of p-AlGaN is equal to 0.7 (0.02 Ω/cm^2^). Hence an asymmetric design is more superior. Moreover, for p-AlGaN/i-In_0.2_Ga_0.8_N/n-Al_0.5_Ga_0.5_N TJ with high Al composition (≥0.5) in the n-AlGaN layer, which is important in deep UV-LEDs to avoid light absorption from the active layer, p-Al_0.7_Ga_0.3_N/i-In_0.2_Ga_0.8_N/n-Al_0.5_Ga_0.5_N has 25.2% lower TJ resistance compared to conventional p-Al_0.5_Ga_0.5_N/i-In_0.2_Ga_0.8_N/n-Al_0.5_Ga_0.5_N TJ. Thus, applying the asymmetric tunnel junction into the UV-LED could lead to a reduction of the tunnel resistance, and the high Al composition p-layer could also adjust the electron blocking effect without additional light absorption due to its larger bandgap. As this might be the first work discussing III-nitride asymmetric tunnel junction, we believe that there is still huge potential to optimize the asymmetric III-nitride tunnel junction with different materials and grading designs.

To better understand the asymmetric TJ designs, band diagrams of p-AlGaN/i-In_0.2_Ga_0.8_N/n-Al_0.3_Ga_0.7_N are shown in Figure 5b. When the Al composition in p-AlGaN is equal to 0.7, the valence band across the zero-energy level in the tunneling region indicated a high tunneling possibility. The tunneling probability can be described by the WKB approximation [10,33].

(5) $${\;T}_{n}=\exp\left\{-2\int_{0}^{x_{n}}\left[\sqrt{\frac{m_{e}^{*}q^{2}N_{D}t^{2}}{\hbar^{2}\varepsilon }}\right]\mathrm{dt}\right\}{,\;x}_{n}=\sqrt{\frac{2\varepsilon \Delta E_{C}}{q^{2}N_{D}}}$$

(6) $$T_{p}=\exp\left\{-2\int_{0}^{x_{p}}\left[\sqrt{\frac{m_{h}^{*}q^{2}N_{D}t^{2}}{\hbar^{2}\varepsilon }}\right]\mathrm{dt}\right\},{\;x}_{p}=\sqrt{\frac{2\varepsilon \Delta E_{V}}{q^{2}N_{D}}}\;$$

(7) $${\;T}_{\mathrm{InGaN}}=\exp\left\{-2\int_{0}^{\frac{E_{g}\varepsilon }{q\sigma }}\left[\sqrt{\frac{{2m}_{\mathrm{InGaN}}^{*}\left({\left(\frac{E_{g,\mathrm{InGaN}}}{2}\right)}^{2}-{\left(\frac{E_{g,\mathrm{InGaN}}}{2}-\frac{E_{g,\mathrm{InGaN}}}{\left(\frac{E_{g}\varepsilon }{q\sigma }\right)}\right)}^{2}\right)}{\hbar E_{g,\mathrm{InGaN}}}}\right]\mathrm{dt}\right\}$$

(8) $$T_{\mathrm{net}}{=T}_{n}{*T}_{p}{*T}_{\mathrm{InGaN}}$$

T_InGaN_, T_n_ and T_p_ are the tunneling probabilities in i-InGaN, n-AlGaN and p-AlGaN layers. $\Delta E_{C}$ and $\Delta E_{V}$ represent the band discontinuities across the n-AlGaN/i-InGaN and i-InGaN/p-AlGaN interfaces. N_A_ and N_D_ are the acceptor and donor doping density, $m_{e}^{*}$ $and{\;m}_{h}^{*}$ are the effective mass of electron and hole. ε is the permittivity and σ  is the induced polarization charge density. ε is the permittivity and σ  is the induced polarization charge density.

From the equations above, the tunneling probability across the devices is determined by both the band discontinuity and the induced polarization charge density. With the increasing Al composition of the p-AlGaN layer, the band discontinuity $\Delta E_{V}\;$ becomes larger, which decreases the tunneling probability. However, the induced polarization is increased according to the first-principal calculation [34], and the larger polarization charge σ  assists the tunneling effect. The factors competing with each other; and in our case, the larger polarization-induced charge with an increase in Al composition in the p-AlGaN layer is more significant than the band offset increment, which leads to a reduction in TJ resistance.

## 6. Summary

In this work, we developed an efficient model for III-nitride TJ resistance prediction to instruct the TJ device design. We constructed the first ML model for p-AlGaN/i-InGaN/n-AlGaN TJ resistance prediction. Our ML model is based on the XG-Boost algorithm and trained with the data calculated by TCAD simulations, which rapidly predicted the p-AlGaN/i-InGaN/n-AlGaN TJ resistances with accuracy as high as 90.5% We compared our prediction with the previous report which shows good agreement. Furthermore, 22,254 combinations of p-AlGaN/i-InGaN/n-AlGaN TJ configurations with different Al or In compositions and InGaN layer thicknesses were investigated by our ML model, and the predicted TJ resistances are shown for future design instruction. Moreover, we demonstrated the effectiveness of the asymmetric tunnel junction design based on our TJ resistance prediction database. By increasing the Al composition in the p-AlGaN layer of the p-AlGaN/i-In_0.2_Ga_0.8_N/n-Al_0.3_Ga_0.7_N TJ structure, the increased polarization charge enhances the tunneling effect, which leads to a significant reduction of the TJ resistance.

## Data Availability

The data presented in this study are available on request from the corresponding author.
